# Monitoring and Measuring Autophagy

**DOI:** 10.3390/ijms18091865

**Published:** 2017-08-28

**Authors:** Saori R. Yoshii, Noboru Mizushima

**Affiliations:** 1 Focal Area Infection Biology, Biozentrum, University of Basel, Klingelbergstrasse 50/70, 4056 Basel, Switzerland; saori.yoshii@unibas.ch; 2 Department of Biochemistry and Molecular Biology, Graduate School and Faculty of Medicine, The University of Tokyo, 7-3-1, Hongo, Bunkyo-ku, Tokyo 113-0033, Japan

**Keywords:** autophagosome, autophagic flux, isolation membrane, Keima, LC3, p62/SQSTM1

## Abstract

Autophagy is a cytoplasmic degradation system, which is important for starvation adaptation and cellular quality control. Recent advances in understanding autophagy highlight its importance under physiological and pathological conditions. However, methods for monitoring autophagic activity are complicated and the results are sometimes misinterpreted. Here, we review the methods used to identify autophagic structures, and to measure autophagic flux in cultured cells and animals. We will also describe the existing autophagy reporter mice that are useful for autophagy studies and drug testing. Lastly, we will consider the attempts to monitor autophagy in samples derived from humans.

## 1. Introduction

Autophagy is an intracellular system that degrades cytosolic proteins and organelles. Autophagy can be categorized into three groups: macroautophagy, microautophagy and chaperon-mediated autophagy (CMA) [1]. Macroautophagy is most widely investigated and best known among the three pathways, and we will focus on the methods to study macroautophagy in the present article. In microautophagy, the lysosome or vacuole itself engulfs a portion of cytoplasm either by invagination of the lysosomal membrane or protrusion of the membrane to surround the cytosol or organelles [2,3]. Microautophagy has been relatively well studied in yeasts but it is still largely obscure in mammalian cells partly because its study relies mostly on morphological analysis by electron microscopy. CMA was observed only in mammalian cells; in CMA, proteins are unfolded and directly translocated into lysosomes dependently on the Lys-Phe-Glu-Arg-Gln (KFERQ)-like motif of the substrate, and on cytosolic heat shock-cognate chaperone of 70 kDa (HSC70), lysosome-associated membrane protein type-2A (LAMP2A) and lysosome resident HSC70 [4]. Methods to monitor chaperon-mediated autophagy have been described elsewhere [5].

When macroautophagy (hereafter simply referred to as “autophagy”) is induced, an isolation membrane encloses a portion of cytoplasm, forming a characteristic double-membraned organelle termed the autophagosome. The autophagosome then fuses with the lysosome to form an autolysosome, the contents of which are then degraded by lysosomal enzymes. Autophagic degradation of cytoplasmic components is essentially non-selective, but can also be selective. Non-selective (bulk) autophagy is important for starvation adaptation, whereas selective autophagy may be more important for maintaining homeostasis of cytosolic proteins and organelles, although these two categories are not mutually exclusive [1]. Autophagy has also been implicated in pathological conditions including neurodegenerative diseases, cancer, and inflammatory diseases [1,6]. Modulation of autophagy has become a potentially interesting therapeutic target in human diseases.

Autophagy can be monitored by two different approaches: (1) direct observation of autophagy-related structures and their fate; and (2) quantification of autophagy-/lysosome-dependent degradation of proteins and organelles. Static analysis of autophagic structures at a certain time point often leads to inaccurate interpretations. To accurately estimate autophagic activity, it is essential to determine autophagic flux, which is defined as the amount of autophagic degradation. Monitoring autophagic flux remains complicated even in cultured cells and model organisms, and is currently unfeasible in humans. Here, we describe currently available methods to detect autophagic structures and to monitor autophagic flux, and discuss the considerations involved in observing autophagic activity in cells, animals, and humans. Autophagy methods have been extensively reviewed [7,8,9,10,11,12,13,14,15,16].

## 2. Identification of Autophagic Structures

### 2.1. The Site of Autophagosome Formation and the Omegasome

**Figure 1:**
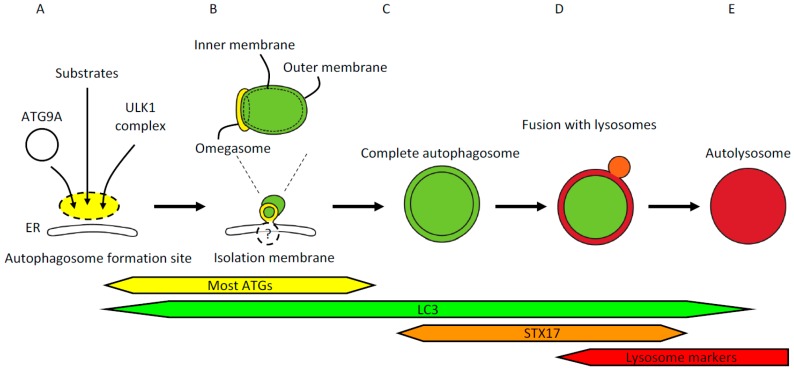
(**A**) The site of autophagosome formation. Upstream autophagy factors and autophagy substrates accumulate at the autophagosome formation site independently from one another. The most upstream autophagy factors are ATG9A in vesicles and the ULK1 complex. Autophagy substrates include p62/SQSTM1, ferritin, and damaged mitochondria. The autophagosome formation site localizes in close apposition to the endoplasmic reticulum (ER); (**B**) Isolation membrane formation. Isolation membranes emerge from within the ring-shaped structure called the omegasome. The omegasome is a phosphatidylinositol 3-phosphate -rich ER subdomain characterized by the existence of double-FYVE domain-containing protein 1 (DFCP1). ATG factors commonly used to visualize isolation membranes include ULK1, WIPI1/2, ATG5, and LC3. Most ATG factors but not LC3 homologs detach from the isolation membranes before completion of autophagosomes. These isolation membrane markers are localized on the outer membrane, whereas LC3 and its homologs and associating p62/SQSTM1 are localized on both outer and inner membranes. Isolation membranes are observed by electron microscopy as crescent-shaped structures that are often flanked by rough ER; (**C**) Completion of autophagosome formation. Several minutes after detachment of ATG factors, LC3-positive autophagosomes acquire syntaxin17 (STX17). STX17 localizes to ER, mitochondria, and complete autophagosomes; therefore, not all the STX17 signals represent autophagosomes. Cytoplasmic components enclosed in the characteristic double-membraned structures mark autophagosomes observed by electron microscopy (EM). The width of the cleft between the outer and inner membranes can vary due to experimental procedures such as fixation; (**D**) Fusion with lysosomes. Autophagosomes that have acquired STX17 fuse with lysosomes to degrade the contents of the autophagosomes. Upon fusion with lysosomes, the space between the outer and inner autophagosomal membranes becomes acidified, followed by collapse of the inner membrane; (**E**) Autolysosome. Fusion of autophagosomes and lysosomes generates autolysosomes that contain degraded cytoplasmic components. Autolysosomes can be identified by EM or colocalization of LC3 and lysosomal markers by fluorescence microscopy.

Autophagy substrates and upstream autophagy factors translocate independently to the autophagosome formation site [17] (Figure 1A). The most upstream autophagy factors are the ULK1 complex, consisting of ULK1, ATG13, FIP200, and ATG101, and ATG9A on vesicles [18]. These upstream autophagy factors then recruit the phosphatidylinositol 3-kinase (PI3K) complex that generates phosphatidylinositol 3-phosphate (PI3P) on the ring-shaped endoplasmic reticulum (ER) structure termed the omegasome, and on the isolation membrane (also known as the phagophore). The omegasome is characterized by the existence of double-FYVE domain-containing protein 1 (DFCP1), from which the isolation membranes protrude [19] (Figure 1B). The ULK1 complex, ATG9A, and the PI3K complex are required for initiation of autophagy; inhibition or deletion of these factors results in no isolation membrane formation, and accumulation of autophagy substrates such as p62, damaged mitochondria, and ferritin at the autophagosome formation site [17,20].

### 2.2. The Isolation Membrane and Autophagosome

PI3P synthesized by the PI3K complex in turn recruits the PI3P-binding WD repeat domain phosphoinositide-interacting (WIPI) family proteins (typically WIPI2) and ATG2A/B, which are required for isolation membrane formation and elongation [20,21,22]. Isolation membranes can be visualized using a component of the ULK1 complex, PI3K complex, WIPI1/2, or autophagy conjugation systems (ATG12 and ATG8/LC3) (Figure 1B). Commonly used markers include ULK1, WIPI1 (which is not necessary for autophagy but can be used as a marker), WIPI2, ATG5, and microtubule-associated protein light chain 3 (LC3). All of these ATG factors, but not LC3 homologs, detach before (or immediately after) the completion of the autophagosome. Then, immediately before or after closure of the edge of the autophagosome, syntaxin17 (STX17) is recruited to the ATG-negative, LC3-positive autophagosome [23] (Figure 1C). Full-length STX17 or STX17 that lacks the entire N-terminal region upstream of the transmembrane domain (STX17TM) serves as a marker for complete (closed) autophagosomes. It should be noted, however, that STX17 also localizes to ER and mitochondria; therefore, not all STX17 signals represent complete autophagosomes.

### 2.3. Fusion with the Lysosome

Mature autophagosomes that have acquired STX17 fuse with the lysosome to degrade their contents [23] (Figure 1D). Fusion between autophagosomes and lysosomes can be visualized using LC3 or STX17, and LysoTracker or LAMP1/2 as markers for autophagosomes and lysosomes, respectively. Immediately after fusion, LysoTracker signals can be observed as a ring-shaped structure that represents an early autolysosome with an intact inner membrane [24] (Figure 1D,E). Several minutes later, the inner membrane is degraded, making the lumen of the autolysosome become LysoTracker positive. Autophagy substrates are finally degraded by lysosomal enzymes. Defects in lysosomal enzymes can result in accumulation of autophagic structures and longer retention of undegraded substrates [25].

### 2.4. Identification of Autophagic Structures by Electron Microscopy

Existence of the cytoplasmic components enclosed in double-membraned vesicles is the hallmark of and provides definitive evidence for autophagosomes [12]. The morphology of the characteristic double membrane of the autophagosomes, however, can greatly differ depending on the conditions and/or the fixatives used [26,27]. The conventional method for electron microscopy results in clear double-membraned structures, easily identified as autophagosomes, with a wide cleft between the outer and inner membranes although the wide opening is likely an artifact due to the fixation method. Autolysosomes are single-membraned structures containing degraded cytoplasmic components. Identification of autophagic structures and examples of misinterpretation have been reviewed in detail [12,13,28].

Morphology of each step of autophagosome formation, using cells deficient in various ATG factors, has been described in detail in cultured cells [20]. However, identification of autophagic structures in tissues by electron microscopy is still a challenging task due to suboptimal fixation, complex three-dimensional organization of the organ, and diverse structures of cellular contents in each tissue and cell type. Though the quality is somewhat limited, immune-electron microscopy, for instance against green fluorescent protein (GFP) in tissues from GFP-LC3 mice (described below), can be used to confirm the existence of autophagic structures in vivo [29].

## 3. Measurement of Autophagic Flux in Cultured Cells

Most of assays described below use LC3 as a model substrate to measure autophagic flux. After translation, the preform of LC3 is cleaved after the C-terminal glycine by ATG4 family proteins to form LC3-I and subsequently conjugated to phosphatidylethanolamine (PE) to form LC3-II. Conjugation of LC3 and other homologs such as γ-aminobutyric-acid-type-A-receptor-associated proteins (GABARAPs) with PE requires the autophagy conjugation systems: the ATG12 (ATG5, ATG7, ATG10, ATG12, ATG16L1) and LC3/GABARAP systems (LC3/GABARAP family proteins, ATG3 and ATG7) [30].

### 3.1. Detection of Autophagic Flux by Immunoblotting Using LC3-II and p62 as Indicators

**Figure 2:**
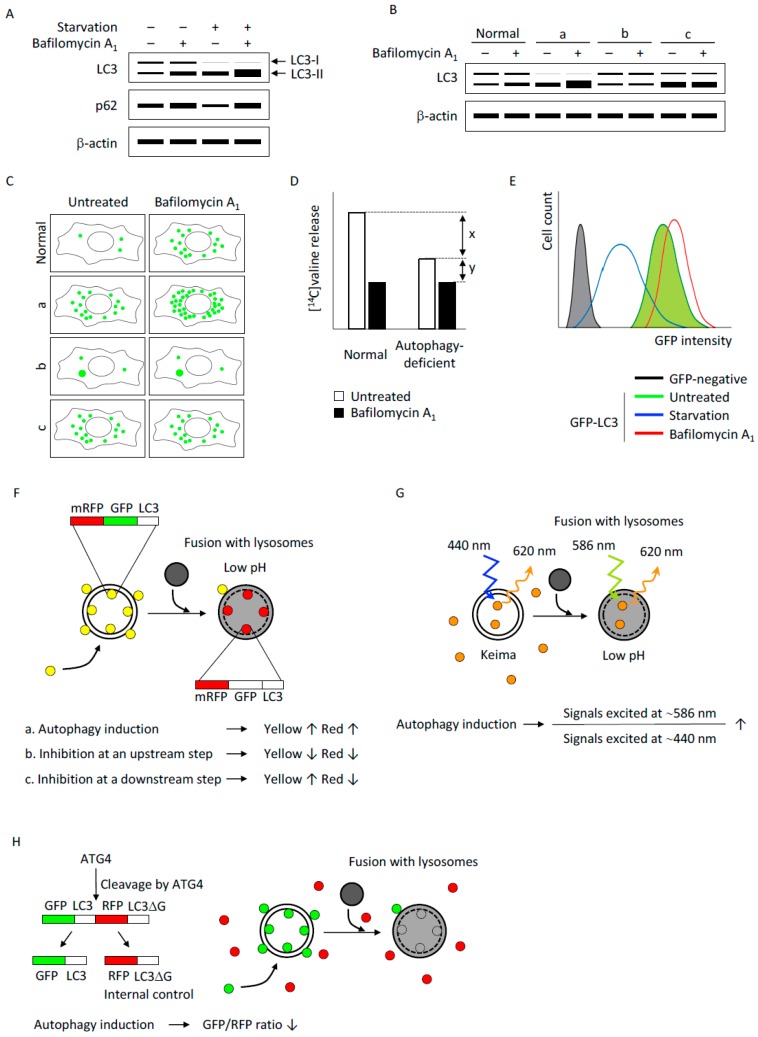
(**A**) A schematic illustration of detection of autophagic flux by immunoblotting. Both LC3-II and p62 are degraded by autophagy; therefore, the amounts of LC3-II and p62 degraded by autophagy, but not their expression levels, provide an estimate of the autophagic activity. Typically, autophagy induction, for instance by nutrient starvation, converts LC3-I to LC3-II and induces an increase in LC3-II and a concurrent decrease in p62. Inhibition of lysosomal degradation by bafilomycin A_1_ causes accumulation of LC3-II and p62, and this increment reflects the amount of LC3-II and p62 that would have been degraded by autophagy over the treatment period; (**B**) A schematic illustration of the interpretation of LC3 immunoblotting results. Autophagic flux in normal cells is estimated by an increase in the LC3-II amount under bafilomycin A_1_ treatment. Group “a” has an increased amount of LC3-II in the basal state, which further increases in the presence of bafilomycin A_1_, suggesting increased autophagic flux in these cells. Group “b” has a low level of LC3-II under basal conditions and does not respond to bafilomycin A_1_ treatment, suggesting that autophagy is suppressed in these cells. Group “c” has a large amount of LC3-II under basal conditions, which does not increase by bafilomycin A_1_ treatment, suggesting decreased autophagic flux (e.g., by a defect in lysosomal degradation) rather than induction of autophagy in these cells; (**C**) Detection of autophagic flux by fluorescence microscopy. LC3 is recruited to autophagosomes forming punctate structures as indicated by green dots. The number of LC3-positive puncta increases in the presence of bafilomycin A_1_ in normal cells. Cells with activated autophagy (group “a”) have a larger number of LC3 puncta, which further increases with bafilomycin A_1_ treatment. Cells that are defective in autophagy induction (group “b”) have a small number of LC3 puncta in basal conditions, and the number does not increase by bafilomycin A_1_ treatment. It should be noted that protein aggregates in these autophagy-deficient cells are also observed as puncta positive for LC3 as well as p62. Cells that are deficient for lysosomal degradation (group “c”) have many LC3-positive puncta under basal conditions but the number of these puncta does not increase by bafilomycin A_1_ treatment as observed in normal cells; (**D**) Measurement of degradation of long-lived proteins labeled with radioisotopes. Degradation of long-lived proteins is detected by the release of isotope-labeled amino acids. The release is inhibited by lysosomal inhibitors (e.g., bafilomycin A_1_). Autophagy-deficient cells have a reduced release of isotope-labeled amino acids without lysosomal inhibitors. The amount indicated by “x” reflects autophagy-dependent protein degradation; the amount indicated by “y” reflects autophagy-independent protein degradation by the lysosome; (**E**) GFP-LC3 degradation assay by flow cytometry. GFP-LC3 on the inner autophagosomal membrane is degraded by the lysosome along with the autophagosome contents; therefore, a decrease in GFP-LC3 over time provides an estimate of the autophagic flux. Degradation of GFP-LC3 is inhibited by bafilomycin A_1_ treatment; (**F**) mRFP-GFP-LC3 tandem fluorescent probe. In the lysosome, the fluorescence of GFP is quenched due to its low pH whereas that of mRFP is stable. Formation of autophagosomes causes an increase in the number of GFP-positive/mRFP-positive (yellow) puncta, and the puncta become GFP-negative /mRFP-positive (red) upon fusion with lysosomes. Autophagy induction results in the increase in both yellow and red puncta, inhibition of autophagy induction results in a decrease in both yellow and red puncta, and inhibition of lysosomal acidification or lysosome fusion results in an increase in yellow puncta and a decrease in red puncta; (**G**) Keima, a fluorescent protein with bimodal excitation. Keima, a fluorescent protein with an emission peak at 620 nm, has bimodal excitation spectra dependent on the surrounding pH. In neutral pH, Keima has an excitation peak at 440 nm, and in the acidic compartment its excitation peak shifts to 586 nm. Therefore, the amount of Keima delivered to the lysosome over time can be estimated by the ratio of signal strength excited at 550 nm divided by that excited at 438 nm. Delivery of cytosolic Keima to the lysosome reflects non-selective (bulk) autophagy, and delivery of Keima fused to a specific protein (such as mitochondria-targeted Keima) reflects selective autophagy. H. GFP-LC3-RFP-LC3ΔG, a fluorescent probe that releases an internal control. The C-terminus of LC3 is cleaved by ATG4, thus one GFP-LC3-RFP-LC3ΔG tandem protein is cleaved into two equimolar proteins, GFP-LC3 and RFP-LC3ΔG. GFP-LC3 is conjugated to phosphatidylethanolamine (PE) and degraded by autophagy. Conversely, RFP-LC3ΔG cannot be conjugated to PE and remains cytosolic; therefore, autophagic flux can be estimated by a decrease in GFP fluorescence in comparison to RFP fluorescence (decrease in the GFP/RFP ratio).

LC3 is conjugated to PE to form LC3-II, which is localized to isolation membranes and autophagosomes (on both outer and inner membranes) [31]. LC3 is currently the most widely used autophagosome marker because the amount of LC3-II reflects the number of autophagosomes and autophagy-related structures. The number of autophagosomes increases by nutrient starvation (deprivation of serum and/or amino acids from cell culture medium), and accordingly the amount of LC3-II also increases. It should be noted, however, that the LC3-II amount at a given time point does not necessarily estimate the autophagic activity, because not only autophagy activation but also inhibition of autophagosome degradation greatly increases the amount of LC3-II. Also, LC3-II can ectopically localize on non-autophagosome structures that are not turned over in the lysosome [8,15,32]. To measure the autophagic flux, it is essential to determine how much LC3-II (as a model substrate of autophagy) is degraded in a lysosome-dependent manner during a certain time period (Figure 2A,B). Degradation of p62 is another widely used marker to monitor autophagic activity because p62 directly binds to LC3 and is selectively degraded by autophagy [33,34] (Figure 2A).

In order to determine the lysosome-dependent degradation, bafilomycin A_1_, a potent V-ATPase inhibitor, or lysosomal enzyme inhibitors such as E64d and pepstatin A are commonly used. The difference in the amount of LC3-II between samples with and without these lysosome inhibitors represents the level of autophagic flux. Antibodies tend to have greater affinity for LC3-II, thus, the signal ratio of LC3-I and LC3-II does not reflect the amount ratio of cytosolic and membrane-bound LC3 [15]. Furthermore, LC3-I can appear very faint depending on the antibodies and cell types. Therefore, LC3-I amount is unreliable to be used as a denominator for quantification of LC3-II (LC3-II/LC3-I). Instead, it is highly recommended to compare the amount of LC3-II with one of the housekeeping proteins, such as tubulin or β-actin, for quantification [9]. The immunoblotting-based method to monitor autophagic flux is described elsewhere [9].

### 3.2. The Number of LC3 Puncta Determined by Fluorescence Microscopy

Although LC3 is a good marker for autophagic structures, it is necessary to measure the “flux” of LC3 puncta, as in the immunoblotting-based assay described above. The increase in the number of LC3 puncta in the presence of a lysosome inhibitor, compared to that in the absence of the inhibitor, represents the number of autophagosomes that would have been degraded during the treatment period (Figure 2C). This is particularly important when cells express aggregate-prone proteins because LC3 can be incorporated into protein aggregates appearing as prominent puncta, which may be mistakenly recognized as autophagic structures.

### 3.3. Degradation of Long-Lived Proteins by the Lysosome

The methods described above measure the degradation of autophagy-specific substrates. Bulk (non-specific) protein degradation by autophagy can be quantified by measuring degradation of long-lived proteins by the lysosome [35] (Figure 2D). Cells are incubated with isotope-labeled amino acids, such as [^14^C]valine, for longer than 10 h (typically 24 h) to ensure labeling of proteins with slow turnover, followed by incubation in the presence of excess unlabeled amino acids for several hours. This incubation period ensures washout of isotope-labeled free amino acids and turnover of short-lived proteins while avoiding re-incorporation of isotope-labeled amino acids derived from degradation. Then, cells are treated with autophagy-inducing stimuli in the presence or absence of lysosome inhibitors. Released isotope-labeled amino acids can be detected in the supernatant after protein precipitation by trichloroacetic acid [10]. It should be noted that this assay is not specific to autophagy but rather measures lysosome-dependent degradation of any proteins including plasma membrane proteins (via endocytosis) and cytosolic proteins and organelles (via the multivesicular body formation pathway, microautophagy or CMA) (Figure 2D). A non-isotopic method to measure bulk protein degradation has also been developed [36].

### 3.4. GFP-LC3 Degradation Determined by Flow Cytometry

Flow cytometry is a potent tool to quantitatively measure the levels of fluorescence-labeled proteins. As mentioned above, LC3 on the inner membrane of the autophagosome is degraded in the lysosome; therefore, the amount of overall LC3 degradation, which can be detected by the decrease in GFP-LC3 fluorescence, is a good indicator of autophagic activity [37] (Figure 2E). The GFP intensity is reduced by nutrient starvation, whereas it is increased by autophagy or lysosome inhibitors.

### 3.5. mRFP-GFP-LC3 Tandem Fluorescent Protein Quenching Assay

The fluorescence of GFP (pKa = 5.9) is quenched in acidic compartments, whereas that of mRFP (pKa = 4.5) is relatively stable and retained even within lysosomes (pH 4–5). Taking advantage of the differential sensitivity to lysosomal acidity, mRFP-GFP-LC3 tandem fluorescent-tagged LC3 can be used to visualize transition from neutral autophagosomes to acidic autolysosomes [38]. mRFP-GFP-LC3 emits both green and red fluorescence signals when the protein localizes to autophagosomes; this is often shown as yellow signals in merged pictures (Figure 2F). Conversely, the fluorescence of GFP but not of mRFP is quenched within autolysosomes making autolysosomes appear red (Figure 2F). Therefore, autophagic flux can be visualized using this single probe; autophagy induction results in increased numbers of both yellow and red puncta whereas inhibition of autophagy at a late step (inhibition of autophagosome maturation or fusion with the lysosome) results in an increase in the number of yellow puncta and a concurrent decrease in red puncta. Detection of RFP-only autolysosomes depends not only on quenching of GFP but also on the stability of RFP in the lysosome. Thus, increase of RFP single-positive puncta can be caused by both increased autophagy flux and decreased lysosomal enzyme activity as long as the lysosome acidity is intact. mTagRFP-mWasabi-LC3 is a derivative of this system where the green fluorescence of mWasabi (pKa = 6.5) is quenched more efficiently than enhanced GFP (EGFP) within the lysosome [39].

### 3.6. Keima: A Fluorescent Protein with Bimodal Excitation

Most of the methods to measure autophagic flux utilize proteins that are selectively degraded by autophagy such as LC3 and p62; all of these rely completely on lipidation of LC3 (or other ATG homologs). Keima, a fluorescent protein that exhibits bimodal excitation spectra, has been developed to monitor non-selective bulk autophagy [40]. Keima is resistant to lysosomal proteases, and becomes excited at 440 and 586 nm in neutral and acidic environments, respectively. The fluorescence of dKeima (dimeric Keima), peaking at 660 nm, has a low ratio of excitation at 550/438 nm in the cytosol. Upon induction of autophagy, punctate structures that have a high ratio of excitation at 550/438 nm appear, representing the accumulation of this probe in autolysosomes (Figure 2G). In theory, this system simply reflects the delivery of diffuse cytosolic proteins into lysosomes, thereby measuring bulk autophagy.

Keima can also be used to monitor selective autophagy. For example, mKeima (monomeric Keima) fused to a mitochondria-targeting sequence (mt-mKeima) serves as a mitophagy marker. Upon delivery of mitochondria to lysosomes, the acidic mt-mKeima signal at the excitation of 550 nm increases. It must be emphasized that Keima-based assays cannot be carried out in fixed cells because this assay completely relies on lysosomal acidity.

### 3.7. GFP-LC3-RFP-LC3ΔG: A Fluorescent Probe that Releases an Internal Control

Another new probe, GFP-LC3-RFP-LC3ΔG, is a fusion protein of GFP-LC3 and RFP-LC3, but the C-terminal glycine of the second LC3 is deleted. This protein is translated as one fusion protein, and is subsequently cleaved by endogenous ATG4 proteins at the C-terminal glycine of the first LC3, generating exactly the same number of GFP-LC3 and RFP-LC3ΔG proteins in the cytosol (Figure 2H). Upon induction of autophagy, GFP-LC3 is conjugated to PE and localizes to the autophagosomes followed by quenching and degradation within autolysosomes. By contrast, RFP-LC3ΔG cannot be conjugated to PE, thereby staying in the cytosol serving as an internal control, reflecting how much GFP-LC3 had existed before it was degraded by autophagy [41]. The GFP/RFP ratio precisely quantifies the cumulative GFP-LC3 degradation by autophagy. Autophagy induction results in an increase in GFP-LC3 degradation and reduction in the GFP/RFP ratio (Figure 2H).

### 3.8. The CMA Reporter KFERQ-PS-CFP2

CMA was observed mainly in vitro using isolated lysosomes; in order to observe CMA activity in cultured cells, CMA reporter proteins have been developed. Proteins that have amino acid sequences related to KFERQ can be recognized by the cytosolic chaperone, HSC70, and delivered to the lysosome to be degraded by CMA. A photoconvertible fluorescent protein fused to the KFERQ-motif, KFERQ-PS-CRP2, is delivered to the lysosome under serum deprivation conditions [42]. This delivery is independent of macroautophagy because the PI3K inhibitor 3-MA does not inhibit the delivery, and is indeed dependent on the CMA receptor LAMP2A. KFERQ-PS-CRP2 emits cyan and green fluorescence before and after photoconversion at 405 nm, respectively; thus, existing and newly synthesized proteins can be distinguished. Using this reporter, CMA activity can be monitored by the increase/decrease of the delivery of photo-converted (green) fluorescence to the lysosomes, and/or by the decrease of the fluorescence intensity of photo-converted reporter by fluorescence microscopy. The decrease of fluorescence of the reporter can also be quantitatively analyzed by fluorescence-activated cell sorting. It should be noted that KFERQ-PS-CRP2 can also be degraded by the multivesicular body pathway and this appears to be the main degradation pathway at least in *Drosophila* [43]. A photoactivable mCherry-fused CMA reporter, KFERQ-PA-mCherry, has also been generated [42].

## 4. Monitoring Autophagy in Animals

### 4.1. LC3-II Flux by Immunoblotting

Nutrient starvation by food withdrawal causes autophagy activation in most organs, for instance in the liver, muscle, and pancreas, but not in the brain. An increase in LC3-II level can be detected by immunoblotting similar to the observation in cultured cells [29]. Autophagic flux in vivo can also be estimated using lysosome inhibitors such as chloroquine and leupeptin [44,45]. Colchicine can also be used as it inhibits autophagosome–lysosome fusion while it has lower toxicity compared to chloroquine in vivo [46].

### 4.2. Accumulation of p62 in Tissues

Accumulation of soluble p62 and of p62-positive aggregates are among the best known characteristics of autophagy-deficient tissues [47]. Soluble and aggregated p62 accumulation can be detected by immunoblotting of tissue lysates using Triton X-100-soluble and -insoluble fractions, respectively. p62 aggregates can also be visualized by immunohistochemistry. While an increase or decrease in the amounts of p62 protein and aggregates can reflect a change in autophagic activity, p62 expression is also transcriptionally regulated. Thus, its protein levels are affected by both increased transcription/translation and decreased degradation. For instance, p62 mRNA levels are upregulated in muscles upon exercise, especially in combination with starvation, which can mask its degradation by autophagy even though autophagic flux is increased [48,49]. Therefore, it is essential to measure p62 mRNA in combination with analysis of the amount of protein. Immunoblotting and histochemistry methods to detect p62 accumulation and aggregate formation are discussed in detail elsewhere [16].

### 4.3. GFP-LC3 Mice

GFP-LC3 mice that express exogenous GFP-LC3 in the whole body have been used to monitor autophagy in vivo [29]. In these mice autophagosomes are visualized in tissue cryosections. Autophagy is induced in various tissues within 24 h of starvation, evidenced by the increased number of GFP-LC3 puncta. Sample preparation methods are described elsewhere [14].

GFP-LC3-positive autophagosomes must be carefully distinguished from GFP-LC3-positive aggregates as well as autofluorescence in tissues [14]. To this end, it is important to compare the results from GFP-LC3 mice with those from wild-type (GFP-LC3-negative) mice where autofluorescence, but not GFP-LC3-positive autophagosomes, is still visible. Autofluorescence can also be seen through filters other than GFP whereas GFP-LC3 signals are specific for the GFP channel. Electron microscopy coupled with immunolabeling against GFP as mentioned above helps to distinguish between autophagy-related and other structures.

### 4.4. mRFP-GFP-LC3 Mice

Mice that express mRFP-GFP-LC3 (or mCherry-GFP-LC3) have been generated to estimate autophagic flux by static analysis [50,51,52]. mRFP-GFP-LC3 expressed in cardiomyocytes detected both autophagosome and autolysosome formation in mice starved for 24 h, and after ischemia and reperfusion, suggesting increased autophagic flux [50]. The autophagy probe can also be virally transduced in tissues; mCherry-GFP-LC3 was introduced by the intracerebroventricular injection of adeno-associated viruses in newborn mice, and its expression was observed throughout the nervous system; the increased number of mCherry-positive and GFP-negative puncta was observed upon rapamycin or trehalose injection and spinal cord injury [51]. Mice expressing RFP-EGFP-LC3 in the whole body have been generated and successfully used to observe starvation-induced autophagy in the kidney, and dynamic change in autophagic activity after ischemia–reperfusion injury in the proximal tubules [52]. Theoretically, fixation of tissues results in neutralization of the lysosomes; therefore, the existence of RFP single-positive signals in fixed tissues is due to the resistance of RFP protein against lysosomal proteases rather than quenching of GFP in the acidic compartment.

### 4.5. Mice that Express Mitophagy Reporter Proteins

Mitophagy reporter mice have been developed using the mt-mKeima probe (mt-Keima mice) [53]. In tissues from mt-Keima mice, for instance in the liver, dextran cascade blue (a fluorescent compound that accumulates in late endosomes and lysosomes) colocalizes with the Keima signal excited at 561 nm but not at 458 nm, confirming that the probes are delivered to the lysosome, thus reflecting mitophagy [53]. A study using this mouse model revealed that mitophagic activity is high in the heart and specific cell types in the brain. Also, mitophagy is suppressed by aging, expression of mutant Huntingtin protein, and high-fat diet, whereas it is activated by hypoxia, spontaneous mutations in mitochondrial DNA, and cachexia caused by malignant tumors in a distant region. It is still unclear, however, whether all of the 561 nm-excited Keima signals universally reflect mitophagy in tissues. It should also be noted that freshly dissected tissues from mt-Keima mice must be immediately imaged without fixation because lysosomes lose their acidity after dissection and fixation [40].

Similarly to mCherry-GFP-LC3, which serves as an autophagy marker, mCherry-GFP targeted to mitochondria (by fusing to the mitochondrial targeting sequence of an outer mitochondrial membrane protein FIS1) serves as a mitophagy marker in *mito*-QC mice [54]. Normal mitochondria appear yellow (GFP positive and mCherry positive), and mitochondria that have been delivered to lysosomes appear red (GFP negative and mCherry positive). High mitophagy activity is observed in the heart, skeletal muscle, brain, liver, and proximal tubules of the kidney of adult mice [54]. In late-stage embryos, high mitophagy activity is observed in restricted areas of the heart and glomeruli of the kidney. The existence, selectivity, and frequency of selective mitophagy in vivo (e.g., in vivo observation of PARKIN-dependent mitophagy) may be possible to examine using these mitophagy reporter mice.

### 4.6. GFP-LC3-RFP-LC3ΔG mice

Mice expressing GFP-LC3-RFP-LC3ΔG can be a powerful tool to monitor both basal and induced autophagy, although the currently available mouse line expresses a sufficiently high level of GFP-LC3-RFP-LC3ΔG in only muscles [41]. As explained above, high autophagic activity results in a low GFP/RFP ratio, and vice versa. Therefore, ratiometry of GFP/RFP in each cell or tissue accurately reflects basal autophagic activity. This method shows that type I muscle fibers have a lower autophagic flux than type II fibers. Induction of autophagy can also be detected; muscles from Torin 1-treated zebrafish, fertilized zebrafish, and eggs and muscles from starved mice show reductions in the GFP/RFP ratio. It should be noted that this is currently the only model to quantitatively, at least to some extent, monitor and compare the basal autophagic flux in different tissues and organs. A mouse line that expresses the appropriate levels of GFP-LC3-RFP-LC3ΔG in the whole body is eagerly awaited.

## 5. Concluding Remarks

Each method described above has its strengths and weaknesses. Recently, residual autophagic activity, although significantly lower than in wild-type cells, has been reported in autophagy conjugation system-deficient cells, where abnormal autophagosomes that have lost the autophagosome marker LC3 are still formed and degraded by lysosomes [24,40,55]. Almost all LC3-based assays, including p62-based assays, fail to observe this residual autophagic flux because these assays require LC3-II formation that depends on the autophagy conjugation systems. Conversely, observation of bulk autophagy by cytosolic dKeima or quantification of lysosome-dependent protein degradation by isotope-labeled long-lived proteins may also include degradation of cytosolic proteins through multivesicular body formation or other forms of lysosome-dependent protein degradation. Therefore, we recommend that suitable assays be chosen based on the aim of the study and a combination of independent assays be used to avoid false interpretations.

Detection of autophagy markers such as LC3-II and/or of accumulation of autophagy substrates such as p62 has been attempted using autopsy or biopsy samples from human patients. However, static analyses cannot distinguish between autophagy upregulation and degradation inhibition, and an increased amount of p62 is not necessarily caused by inhibition of autophagy as described above. Furthermore, human samples are often limited in quality due to difficulty in obtaining fresh tissues, thus the data need particularly careful evaluation taking into consideration possible effects caused by sample quality and preparation. Presently, there is no established method to measure autophagic flux in humans; therefore, it remains practically impossible to monitor autophagy properly in humans. Static analyses with the limitations stated above are often misleading, or can provide only suggestive results at best.

Genetic mutations in humans that may cause changes in autophagic flux have been studied using patient-derived cells such as fibroblasts, lymphoblastoid cell lines, or induced pluripotent stem cells differentiated into the affected tissues [25,56,57,58]. Autophagic flux can be tested properly in these cells ex vivo. How much the results using cell types differing from affected tissues, or how much the defects observed ex vivo reflect the pathology of patients and the cause of symptoms remain to be addressed.
